# Reclassifying causes of obstetric death in Mexico: a repeated cross-sectional study

**DOI:** 10.2471/BLT.15.163360

**Published:** 2016-05-02

**Authors:** Margaret C Hogan, Biani Saavedra-Avendano, Blair G Darney, Luis M Torres-Palacios, Ana L Rhenals-Osorio, Bertha L Vázquez Sierra, Patricia N Soliz-Sánchez, Emmanuela Gakidou, Rafael Lozano

**Affiliations:** a University of Washington, Seattle, United States of America (USA).; b National Institute of Public Health, Av. Universidad 655, Col. Santa Maria Ahucatitlan, 62100, Cuernavaca, Morelos, Mexico.; c Secretaría de Salud, Mexico City, Mexico.; d Pan American Health Organization, Washington, USA.

## Abstract

**Objective:**

To describe causes of maternal mortality in Mexico over eight years, with particular attention to indirect obstetric deaths and socioeconomic disparities.

**Methods:**

We conducted a repeated cross-sectional study using the 2006–2013 *Búsqueda intencionada y reclasificación de muertes maternas* (BIRMM) data set. We used frequencies to describe new cases, cause distributions and the reclassification of maternal mortality cases by the BIRMM process. We used statistical tests to analyse differences in sociodemographic characteristics between direct and indirect deaths and differences in the proportion of overall direct and indirect deaths, by year and by municipality poverty level.

**Findings:**

A total of 9043 maternal deaths were subjected to the review process. There was a 13% increase (from 7829 to 9043) in overall identified maternal deaths and a threefold increase in the proportion of maternal deaths classified as late maternal deaths (from 2.1% to 6.9%). Over the study period direct obstetric deaths declined, while there was no change in deaths from indirect obstetric causes. Direct deaths were concentrated in women who lived in the poorest municipalities. When compared to those dying of direct causes, women dying of indirect causes had fewer pregnancies and were slightly younger, better educated and more likely to live in wealthier municipalities.

**Conclusion:**

The BIRMM is one approach to correct maternal death statistics in settings with poor resources. The approach could help the health system to rethink its strategy to reduce maternal deaths from indirect obstetric causes, including prevention of unwanted pregnancies and improvement of antenatal and post-obstetric care.

## Résumé

**Objectif:**

Décrire les causes de mortalité maternelle au Mexique sur une période de huit ans, en s'intéressant particulièrement aux décès dus à des causes obstétricales indirectes ainsi qu'aux disparités socioéconomiques.

**Méthodes:**

Nous avons réalisé une étude transversale répétée à l'aide des données de l'outil *Búsqueda intencionada y reclasificación de muertes maternas* (BIRMM) 2006–2013. Nous nous sommes basés sur les fréquences pour décrire les nouveaux cas, la répartition des causes et la reclassification des cas de mortalité maternelle selon la procédure BIRMM. Nous avons utilisé des tests statistiques pour analyser les différences, sur le plan des caractéristiques sociodémographiques, entre les décès pour cause directe et indirecte, ainsi que les différences dans la proportion globale de décès pour cause directe et indirecte, par année et par niveau de pauvreté des municipalités.

**Résultats:**

Un total de 9043 décès maternels a été examiné. Nous avons constaté une augmentation de 13% du nombre global de décès maternels identifiés (de 7829 à 9043) et la proportion de décès maternels classés comme décès maternels tardifs a été multipliée par trois (passant de 2,1% à 6,9%). Sur la période étudiée, le nombre de décès dus à des causes obstétricales directes a diminué, mais il n'a été observé aucun changement dans le nombre de décès dus à des causes obstétricales indirectes. Les décès pour cause directe étaient concentrés chez les femmes vivant dans les municipalités les plus pauvres. En comparaison des femmes décédées de causes directes, les femmes décédées de causes indirectes avaient eu moins de grossesses et étaient légèrement plus jeunes, plus instruites et plus susceptibles de vivre dans des municipalités plus riches.

**Conclusion:**

La procédure BIRMM est une approche qui permet de rectifier les statistiques sur les décès maternels dans les zones ayant peu de ressources. Cette approche pourrait permettre de repenser la stratégie du système de santé en vue de réduire le nombre de décès maternels dus à des causes obstétricales indirectes, avec notamment la prévention des grossesses non désirées et l'amélioration des soins prénataux et post-obstétricaux.

## Resumen

**Objetivo:**

Describir las causas de la mortalidad materna en México durante ocho años, con especial atención a las muertes obstétricas indirectas y a las desigualdades socioeconómicas.

**Métodos:**

Se realizó un estudio transversal repetido utilizando el conjunto de datos *Búsqueda intencionada y reclasificación de muertes maternas* (BIRMM) de 2006-2013. Mediante el proceso de la BIRMM, se utilizaron frecuencias para describir nuevos casos, las distribuciones de las causas y la reclasificación de los casos de mortalidad materna. Se utilizaron pruebas estadísticas para analizar las diferencias en cuanto a las características sociodemográficas entre las muertes directas e indirectas y las diferencias en el porcentaje del total de muertes directas e indirectas, por año y por nivel de pobreza de los municipios.

**Resultados:**

Un total de 9 043 muertes maternas fueron sujetas al proceso de revisión. En general, el número de muertes maternas identificadas aumentó un 13% (de 7 829 a 9 043) y el porcentaje de muertes maternas clasificadas como muertes maternas tardías se triplicó (de un 2,1% a un 6,9%). Durante el periodo de estudio, las muertes obstétricas directas se redujeron, mientras que las muertes derivadas de causas obstétricas indirectas se mantuvieron sin cambios. Las muertes directas se concentraron en mujeres que vivían en los municipios más pobres. En comparación con aquellas que fallecían por causas directas, las mujeres que fallecían por causas indirectas tuvieron menos embarazos y eran ligeramente más jóvenes, tenían una mejor educación y más posibilidades de vivir en municipios más adinerados.

**Conclusión:**

La BIRMM es un enfoque cuyo objetivo es corregir las estadísticas de mortalidad materna en entornos con pocos recursos. El enfoque podría ayudar al sistema sanitario a reconsiderar su estrategia para reducir las muertes maternas por causas obstétricas indirectas, incluyendo la prevención de embarazos no deseados y la mejora del cuidado prenatal y postobstétrico.

## ملخص

**الغرض:**

وصف أسباب الوفاة بين الأمهات في المكسيك على مدى ثماني سنوات، مع تركيز الاهتمام على الوفاة المرتبطة بالولادة والناتجة عن أسباب غير مباشرة، وعلى الفوارق الاجتماعية الاقتصادية.

**الطريقة أجرينا دراسة متكررة تشمل قطاعات متعددة باستخدام مجموعة البيانات المتوفرة في:**

*Búsqueda intencionada y reclasificación de muertes maternas * (BIRMM) للفترة من 2006 حتى 2013.

واستخدمنا معدلات تكرار ظهور الحالات لوصف الحالات الجديدة، وانتشار أسباب ظهورها، ولإعادة تصنيف حالات الوفاة بين الأمهات، وذلك بالاستعانة بالإجراءات التي يتضمنها BIRMM.

واستخدمنا الاختبارات الإحصائية لتحليل الفروق في الخصائص السكانية الاجتماعية بين حالات الوفاة الناتجة عن أسباب مباشرة وتلك الناتجة عن أسباب غير مباشرة، والفروق في معدلات حالات الوفاة الناتجة عن أسباب مباشرة وتلك الناتجة عن أسباب غير مباشرة بوجه عام، وذلك وفقًا للعام ومستوى الفقر في الإقليم.

**النتائج:**

خضعت حالات الوفاة بين الأمهات بإجمالي 9043 حالة لإجراءات المراجعة. ظهرت زيادة بنسبة 13‏% (من 7829 إلى 9043) فيما تم اكتشافه من حالات الوفاة بين الأمهات بوجه عام، كما ظهرت زيادة بمقدار ثلاثة أضعاف في معدل الوفاة بين الأمهات والمصنفة كحالات للوفاة وقعت بين الأمهات في فترات متأخرة (من 2.1‏% إلى 6.9‏%). وعلى مدى فترة الدراسة تراجعت أعداد الوفيات المرتبطة بالولادة والناتجة عن أسباب مباشرة، بينما لم يظهر أي تغيير في أعداد الوفيات الناتجة عن أسباب غير مباشرة تتعلق بالولادة. وتركزت الوفيات الناتجة عن أسباب مباشرة في النساء ممن يعشن في الأقاليم الأشد فقرًا. وقد تبين أن النساء المتوفيات لأسباب غير مباشرة – مقارنةً بأولئك المتوفيات لأسباب غير مباشرة – قد خضن تجارب حمل أقل عددًا، وكن أصغر سنًا، وحصلن على قدر أكبر من التعليم، كما زاد لديهن احتمال المعيشة في أقاليم تتميز بارتفاع المستوى المادي.

**الاستنتاج:**

يمثل BIRMM أحد الأساليب المناسبة لتصحيح البيانات الإحصائية عن الوفاة بين الأمهات في البيئات التي تفتقر إلى الموارد الكافية. وقد يوفر ذلك الأسلوب المساعدة للقائمين على النظام الصحي لإعادة النظر في الاستراتيجية المتبعة لديهم لخفض معدل الوفاة بين الأمهات والناتجة عن أسباب غير مباشرة تتعلق بالولادة، بما في ذلك الوقاية من حالات الحمل غير المرغوب فيها وتحسين الرعاية المقدَّمة في فترة الحمل وفيما بعد الولادة.

## 摘要

**目的:**

旨在说明八年多内墨西哥境内孕产妇死亡的原因，重点关注间接孕产妇死亡人数和社会经济方面的差异。

**方法:**

我们采用 2006 至 2013 年的 *Búsqueda intencionada y reclasificación de muertes maternas* (BIRMM) 数据集开展了一项重复横断面研究， 并且利用频率分析，通过 BIRMM 方法说明新增案例、原因分布以及孕产妇死亡案例的重新分类情况。 我们采用统计测试，按照年份和地区贫困等级分析了直接与间接死亡人数的社会人口特性差异，以及总体直接死亡人数与间接死亡人数的比例差异。

**结果:**

研究过程中，我们共查阅了 9043 个孕产妇死亡案例。 确认的产妇死亡人数总体增长了 13%（从 7829 例增至 9043 例），归类为孕产妇后期死亡人数的孕产妇死亡人数的比例增至三倍（从 2.1% 增至 6.9%）。 研究期间，直接孕产妇死亡人数有所降低，但是间接孕产妇死亡人数保持不变。 直接死亡人数集中于居住在最贫困地区的女性。 与死于直接原因的女性相比，死于间接原因的女性生育次数更少、年龄更为年轻、受教育程度更高、并且更有可能居住在更富裕的地区。。

**结论:**

BIRMM 是一种在资源贫乏的情况下更正孕产妇死亡统计数据的方法。 该方法可以帮助卫生系统重新思考其降低间接孕产妇原因造成的孕产妇死亡人数的策略。这些策略包括预防意外怀孕以及改善产前和产后护理。

## Резюме

**Цель:**

Описать причины материнской смертности в Мексике за восемь лет, уделяя особое внимание акушерским смертям по непрямым причинам, а также из-за социально-экономического неравенства.

**Методы:**

Было проведено повторное одномоментное поперечное исследование с использованием набора данных Búsqueda intencionada y reclasificación de muertes maternas (BIRMM) 2006–2013 гг. Для описания новых случаев, распределения причин и повторной классификации случаев материнской смертности с помощью процедуры BIRMM использовались данные о частотности. С помощью статистических критериев были проанализированы различия в социально-демографических характеристиках рожениц, умерших по прямым и непрямым причинам, и различия в доле, которую эти смерти составляли в общем количестве смертей по прямым и непрямым причинам, в зависимости от года и уровня бедности муниципального образования.

**Результаты:**

В ходе исследования было изучено 9043 случая смертей среди матерей. Общее количество определенных материнских смертей увеличилось на 13% (с 7829 до 9043), а доля материнских смертей, классифицированных как поздняя материнская смерть, увеличилась в три раза (с 2,1 до 6,9%). На протяжении исследования количество акушерских смертей по прямым причинам снизилось, однако количество смертей по непрямым акушерским причинам не изменилось. Смерть по прямым причинам в большинстве случаев наступала среди женщин, проживавших в беднейших муниципальных образованиях. По сравнению с роженицами, умершими по прямым причинам, женщины, чья смерть наступила по непрямым причинам, имели меньше беременностей и были несколько младше, лучше образованны и с большей вероятностью проживали в более зажиточных поселениях.

**Вывод:**

Процедура BIRMM представляет собой один из подходов к получению правильной статистики по материнской смертности в условиях ограниченности ресурсов. С помощью этого подхода специалисты системы здравоохранения могли бы пересмотреть свою стратегию по сокращению количества материнских смертей по непрямым акушерским причинам, включающую предупреждение нежелательной беременности и повышение качества дородовой и послеродовой помощи.

## Introduction

Maternal mortality – defined as the death of a woman during pregnancy, childbirth or in the 42 days after delivery – is used as an outcome measure for any health system. The indicator of maternal mortality is the maternal mortality ratio (MMR), which is defined as the number of maternal deaths per 100 000 live births. MMR is a problematic indicator to measure, due to the relative few maternal deaths.^1^^–^^5^ It is even more challenging to measure cause-specific maternal mortality, since available methods have either low sensitivity or specificity.^6^ Therefore, accurate estimates of cause-specific maternal mortality are often not available in many countries.^7^

The *International statistical classification of diseases and related health problems, 10th revision* (ICD-10)^8^ manual divides the causes of maternal mortality into three broad categories: direct obstetric deaths (resulting from obstetric complications of the pregnant state); indirect obstetric deaths (resulting from a disease, often pre-existing and aggravated by the physiologic effects of the pregnancy); and late maternal death (death between 42 days and one year post-obstetric event).^8^^,^^9^ Sequelae, which is maternal deaths that occur one year or more after delivery, is included in the late maternal death category. Indirect maternal deaths represent on average 20% of a country’s overall maternal mortality,^10^^–^^12^ but this proportion varies considerably across settings. Particularly indirect and late maternal causes are likely to be misclassified as non-maternal deaths.^6^^,^^13^^,^^14^ Even within countries with a very high quality vital registration system, there is a wide variation in the fraction of maternal deaths attributable to indirect causes; estimates range from zero to more than half of all reported maternal deaths.^13^^–^^22^

In recent decades, Mexico has improved its measurement of maternal mortality, but problems of underreporting and misreporting in its vital statistics systems still exist.^23^^,^^24^ In response, a new strategy was undertaken by the government in 2002 that aimed to identify all maternal deaths in Mexico using an approach of intentional search, review and reclassification of maternal deaths. A new procedure, referred to as *Búsqueda intencionada y reclasificación de muertes maternas* (BIRMM) was put in place,^25^^,^^26^ which provides a mechanism for a comprehensive examination of maternal deaths in Mexico.

There has been a rapid increase in obesity,^27^^,^^28^ diabetes, hypertension, and hypercholesterolaemia in Mexico,^29^^,^^30^ which puts women of reproductive age at higher risk for pre-existing hypertensive disorders and diabetes mellitus. The epidemiologic transition from communicable to noncommunicable diseases has implications for maternal health.

The purpose of this study is to describe the reclassified and newly identified maternal deaths – especially indirect obstetric deaths – identified through the BIRMM process. We also compared sociodemographic characteristics at individual and municipality level of the women who died from direct and indirect causes.

## Methods

We conducted a repeated cross-sectional study using the 2006–2013 BIRMM data set, which includes all deaths in women of reproductive ages (10–54 years). The aim was to identify miscoded maternal deaths. We investigated the underlying cause of death for those who had been assigned to a subset of 46 ICD-10 codes that we suspected of being maternal deaths but did not have maternal codes from the ICD-10 O chapter.^25^ (The list of the 46 ICD codes are available from the corresponding author). In addition to these 46 codes, we also investigated: deaths that were assigned maternal codes found in the ICD-10 O chapter (Table 1); deaths with suspicious or incomplete codes; deaths with complications but without a valid underlying cause of death; and all death certificates where the pregnancy checkbox had been ticked.

**Table 1:** ICD-10 codes for indirect causes of maternal deaths^8^

Category	Sub-category	Title
O10	O10.0–O10.9	Pre-existing hypertension complicating pregnancy, childbirth and the puerperium
O24	O24.0–O24.9	Diabetes mellitus during pregnancy
O98	O98.0–O98.9	Maternal infectious and parasitic diseases classifiable elsewhere but complicating pregnancy, childbirth and the puerperium
O99	O99.0–O99.8	Other maternal diseases classifiable elsewhere but complicating pregnancy, childbirth and the puerperium

ICD: *International classification of diseases and related health problems, 10^th^ revision*.

The BIRMM review process collates maternal mortality and sociodemographic data from available mortality data sources including death certificates, medical records, verbal autopsy records and confidential enquiry or autopsy reports. (The distribution of information contributed by each data source is available from the corresponding author). The collated information was reviewed by two independent coders. In the event of a disagreement when assigning the appropriate code, a senior reviewer examined all documents and assigned the final cause. Full details of the BIRMM protocol have been published elsewhere.^25^

We extracted the following data from the BIRMM: individual-level sociodemographic data (age, marital status, education level, number of pregnancies and number of prenatal visits during the last pregnancy); care-related information on each maternal death (place of care where first complication was documented and where skilled birth attendant care was provided); and place of death. The proportions of missing data for each year for the care-related information are available from the corresponding author. We used cause-of-death data as reported before and after the intentional review process. In addition, we used the 2010 municipality-level development index^31^ as a measure of community level socioeconomic status. The development index data were grouped into quintiles and merged into the individual level BIRMM data record according to the residence municipality of the deceased. To calculate the MMR, live births data for 2006–2013 were obtained from the General Directorate of Health Information.^32^ This study was approved by the ethics committee of the National Institute of Public Health, Mexico.

### Analysis

Table 1 presents the ICD-10 categories and associated sub-categories and titles for codes used for deaths identified as indirect obstetric deaths, as listed in the manual. The list includes all codes that constitute the internationally agreed definition of indirect maternal death. Maternal deaths related to human immunodeficiency virus (HIV) infections were treated as special cases due to evolving ICD-10 coding rules. HIV-related deaths coded to the specific HIV ICD codes (B20-B24) and the recently created HIV ICD code (O98.7) were included and recoded as indirect maternal deaths.

New and recoded indirect maternal deaths, their cause distribution and reclassification by development index quintile were examined using tabulations. We used descriptive statistics to examine differences in direct and indirect cause, before and after the review process and across the eight years (2006–2013). Sociodemographic characteristics and health service use were examined and tested using *t*-test and *χ^2^* tests for differences in the proportion and means of direct and indirect deaths. Maternal mortality cause was analysed by municipality-level development index. All analyses were done in Stata version 13.1 (StataCorp. LP, College Station, United States of America).

## Results

The total number of deaths in women of reproductive age during the study period obtained from the BIRMM was 357 446, of which 9043 deaths (2.5%) were subjected to the maternal death cause review and reclassification process.^33^

Table 2 summarizes the findings from the reclassification process. Before the review 7829 deaths were classified as maternal deaths. Of the deaths coded as non-maternal, we reclassified 1214 (13.4%) to maternal deaths. The number of late maternal deaths increased from 192 (2.1%) before the review to 628 (6.9%) after the review, representing over threefold increase in the proportion of late maternal deaths. There was a 6.8% (from 2099 to 2243 deaths) and 11.4% (from 5538 to 6172 deaths) increase in deaths categorized as indirect and direct, respectively. Deaths that were categorized the same before and after the review were, 85.5% (5281/6172) of direct, 68.8% (1544/2243) of indirect and 28.3 (178/628) of late maternal deaths.

**Table 2:** Summary of the intentional search and review outcomes of maternal deaths, by municipality-level development index quintiles, Mexico, 2006–2013

Cause of death	After review			
	Cause of death, no. (%)			Total, no. (%)
	Indirect^a^	Direct^b^	Late maternal^c^	
**All quintiles**				
Before review				
Non-maternal^d^	479 (21.3)	365 (5.9)	370 (58.9)	1214 (13.4)
Indirect^a^	1544 (68.8)^e^	514 (8.3)	41 (6.5)	2099 (23.2)
Direct^b^	218 (9.7)	5281 (85.5)^e^	39 (6.2)	5538 (61.2)
Late maternal^c^	2 (0.1)	12 (0.2)	178 (28.3)^e^	192 (2.1)
Total	2243 (100.0)	6172 (100.0)	628 (100.0)	9043 (100.0)
**Quintile 1 (poorest)**				
Before review				
Non-maternal^d^	280 (21.1)	193 (6.4)	221 (57.7)	694 (14.7)
Indirect^a^	921 (69.4)^e^	303 (10.1)	22 (5.7)	1246 (26.4)
Direct^b^	124 (9.3)	2507 (83.3)^e^	23 (6.0)	2654 (56.2)
Late maternal^c^	2 (0.2)	5 (0.2)	117 (30.5)^e^	124 (2.6)
Total	1327 (100.0)	3008 (100.0)	382 (100.0)	4718 (100.0)
**Quintile 5 (wealthiest)**				
Before review				
Non-maternal^d^	38 (18.8)	36 (3.3)	34 (64.1)	108 (8.1)
Indirect^a^	134 (66.3)^e^	48 (4.4)	5 (9.4)	187(13.9)
Direct^b^	30 (14.8)	1008 (92.1)^e^	1 (1.9)	1039 (77.0)
Late maternal^c^	0 (0.0)	2 (0.2)	13 (24.5)^e^	15 (1.1)
Total	202 (100.0)	1094 (100.0)	53 (100.0)	1350 (100.0)

^a^ Indirect is defined as maternal deaths resulting from a disease, often pre-existing and aggravated by the physiologic effects of the pregnancy.

^b^ Direct is defined as maternal deaths resulting from obstetric complications during pregnancy.

^c^ Late maternal is defined as death between 42 days and one year post-obstetric event.

^d^ All other maternal deaths not coded to direct, indirect or later maternal.

^e^ Concordant pair before and after review.

In the poorest municipalities, 14.7% (694) of maternal deaths were recoded from non-maternal to maternal, while in the wealthiest municipalities this figure was 8.1% (108). An additional 14.8% (30) of all direct deaths in the wealthiest quintile were reclassified to indirect deaths (Table 2).

Table 3 (available at: http://www.who.int/bulletin/volumes/94/5/15-1633560) shows the original codes of the deaths recoded to indirect maternal deaths. Many indirect maternal deaths were originally misclassified as infectious and parasitic diseases (96), diseases of the circulatory system (94), diseases of the respiratory system (70) and neoplasms (56). In addition, there was miscoding within the maternal chapter of the ICD. For instance, 40 deaths were originally assigned to O10–O16 (oedema, proteinuria and hypertensive disorders in pregnancy, childbirth and the puerperium); 46 deaths were assigned to O21, O23–O31, O34 (other complications of pregnancy) and 46 more maternal deaths were assigned with the codes O89–O92 (other complications during the puerperium). Two hundred and twenty indirect maternal deaths were coded as direct obstetric causes before the correction.

**Table 3:** Cause of death reclassified as indirect maternal deaths, Mexico, 2006–2013

ICD-10 block^8^	Title	No. of recorded cases (%) (*n* = 699)
**Non-maternal**		
A15–B49	Infectious and parasitic diseases	96 (13.7)
C00–C14	Malignant neoplasms of lip, oral cavity and pharynx	1 (0.1)
C15–C96, D10–D48	Other malignant, benign and uncertain or unknown behaviour neoplasms	56 (8.0)
D00–D09	In situ neoplasms (carcinoma)	1 (0.1)
D55–D89	Diseases of blood and blood-forming organs	8 (1.1)
E00–E90	Endocrine, nutritional and metabolic diseases	32 (4.6)
F50–F59	Behavioural syndromes associated with physiological disturbances and physical factors	1 (0.1)
G00–G99	Diseases of the nervous system	21 (3.0)
I00–I99	Diseases of the circulatory system	94 (13.4)
J00–J99	Diseases of the respiratory system	70 (10.0)
K00–K93	Diseases of the digestive system	37 (5.3)
L00–L08	Infections of the skin and subcutaneous tissue	1 (0.1)
M00–M99	Diseases of the musculoskeletal system and connective tissue	7 (1.0)
N00–N99	Diseases of the genitourinary system	9 (1.3)
Q00–Q99	Congenital malformations, deformations and chromosomal abnormalities	20 (2.9)
R10–R19	Symptoms and signs involving the digestive system and abdomen	1 (0.1)
R50–R69	General symptoms and signs	7 (1.0)
R95–R99	Ill-defined and unknown causes of mortality	1 (0.1)
V01–Y98	External causes	16 (2.3)
**Maternal**		
O01	Hydatidiform mole	1(0.1)
O02–O08	Other pregnancy with abortive outcome	9 (1.3)
O10–O16	Oedema, proteinuria and hypertensive disorders in pregnancy, childbirth and the puerperium	40 (5.7)
O20, O45–O46, O67	Premature separation of placenta and other haemorrhage of pregnancy or birth	12 (1.7)
O21, O23–O31, O34	Other complications of pregnancy	46 (6.6)
O22,O87	Other maternal disorders predominantly related to pregnancy	2 (0.3)
O35–O43, O68–O69	Fetal distress and other complications of pregnancy or birth	16 (2.3)
O47–O48, O60–O75	Prolonged pregnancy, other complications of labour	9 (1.3)
O72	Postpartum haemorrhage	6 (0.9)
O88	Obstetric embolism	12 (1.7)
O89–O92	Other complications during the puerperium	46 (6.6)
O96–O97	Late and sequelae maternal death	2 (0.3)
A34, O85–O86	Obstetrical tetanus, complications predominantly related to the peurperium	19 (2.7)

**Fig. 1:**
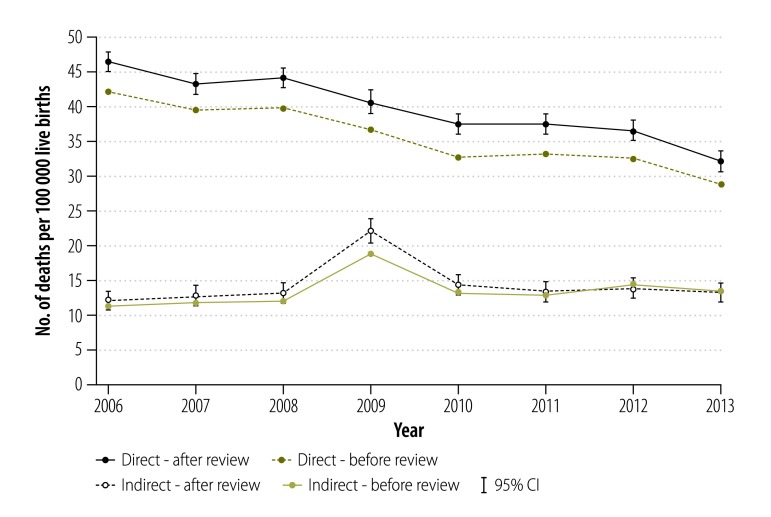
Direct and indirect maternal deaths before and after the review process, Mexico, 2006–2013

For maternal deaths categorized as direct, there was a declining trend in MMR between 2006 and 2013, from 46.4 to 32.1 deaths per 100 000 live births. There was no change for indirect maternal deaths. MMR for indirect deaths was 12.2 deaths per 100 000 live births in 2006 and 13.3 deaths per 100 000 live births in 2013. There was a peak in 2009, mainly due to the Influenza A (H1N1) epidemic, which is known to increase the risk of hospitalization, severe illness and death in pregnant women.^34^^,^^35^ The trends for both direct and indirect causes were similar before and after reclassifying the causes of deaths (Fig. 1).

CI: confidence interval.

Comparison of sociodemographic characteristics and health system use showed that women who died of indirect maternal causes had fewer pregnancies, were slightly younger and were better educated than women dying of direct causes. The deceased women were also more likely to have delivered, received care for their first complication and died in *Instituto Mexicano del Seguro Social* facilities, which are employment-based insurance-affiliated facilities. Skilled birth attendants were more likely to have been present for the mothers who died of direct causes, but this could be due to the fact that women who died of indirect causes died before delivery (Table 4; available at: http://www.who.int/bulletin/volumes/94/5/15-1633560).

**Table 4:** Characteristics of indirect and direct maternal deaths, Mexico, 2006–2013

Characteristics	Cause of death	
	Indirect	Direct
**Number of pregnancies (mean, SD)**	2.45 (0.04)	3.03 (0.03)*^,a^
**Age (mean, SD)**	27.40 (0.14)	28.70 (0.09)*^,a^
**Number of prenatal visits (mean, SD)**	3.26 (0.07)	2.86 (0.04)*^,a^
**Marital status, *n***	2208	6022
Single, *n* (proportion)	394 (0.18)	900 (0.15)
Common law, divorced or widowed, *n* (proportion)	869 (0.39)	2415 (0.40)
Married, no. (%)	945 (0.43)	2707 (0.45)*^,b^
**Education, *n***	2131	5807
Primary, no. (%)	849 (0.40)	2660 (0.46)
Secondary, no. (%)	726 (0.34)	1851 (0.32)
High school or more, no. (%)	557 (0.26)	1296 (0.22)*^,b^
**Place of death, *n***^4^	1732	4331
Secretaria de Salud, no. (%)	903 (0.52)	2143 (0.49)
IMSS/ISSSTE/SEDENA, no. (%)	511 (0.30)	1010 (0.23)
Private medical unit, no. (%)	92 (0.05)	410 (0.09)
Home, street, other, no. (%)	226 (0.13)	768 (0.18)*^,b^
**Place of delivery, *n***	1657	5329
Secretaria de Salud, no. (%)	901 (0.54)	2603 (0.49)
IMSS/ISSSTE/SEDENA, no. (%)	491 (0.30)	1048 (0.20)
Private medical unit, no. (%)	150 (0.09)	833 (0.16)
Home, street, other, no. (%)	115 (0.07)	845 (0.16)*^,b^
**Place of care for first complication, *n***	1875	5129
Secretaria de Salud, no. (%)	1044 (0.56)	2864 (0.56)
IMSS/ISSSTE/SEDENA, no. (%)	533 (0.28)	1069 (0.21)
Private medical unit, no. (%)	285 (0.15)	1104 (0.22)
Home, street, other, no. (%)	13 (0.01)	92 (0.02)*^,b^
**Development index, (municipality), *n***	2237	6145
Quintile 1 – Poorest, no. (%)	202 (0.09)	1094 (0.18)*^,b^
Quintile 2, no. (%)	194 (0.09)	666 (0.11)
Quintile 3, no. (%)	197 (0.09)	604 (0.10)
Quintile 4, no. (%)	317 (0.14)	773 (0.13)
Quintile 5 – Wealthiest, no. (%)	1327 (0.599	3008 (0.49)
**Skilled birth attendant, *n***	1910	5477
Doctor, no. (%)	1361 (0.71)	4267 (0.78)
Nurse/auxiliary/midwife, no. (%)	43 (0.02)	460 (0.08)
Relative/other, *n* (proportion)	20 (0.01)	231 (0.04)
No delivery, *n* (proportion)	486 (0.25)	519 (0.09)

IMSS: Instituto Mexicano del Seguro Social, ISSSTE: Instituto de Seguridad y Servicios Sociales de los Trabajadores del Estado, SEDENA: Secretaría de la Defensa Nacional, SD: standard deviation.

* *P* < 0.05; **P < 0.01.

^a^ *Tested using differences in mean*

^b^ *Tested *using an overall Pearson χ^2^.

**Fig. 2:**
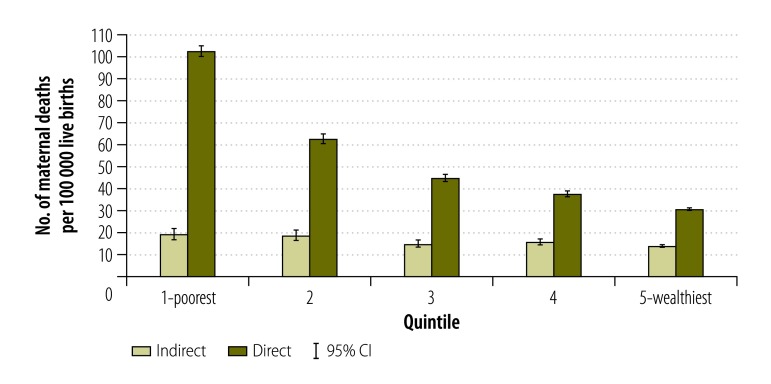
Direct and indirect maternal deaths by municipality-level development index, Mexico, 2006–2013

**Fig. 3:**
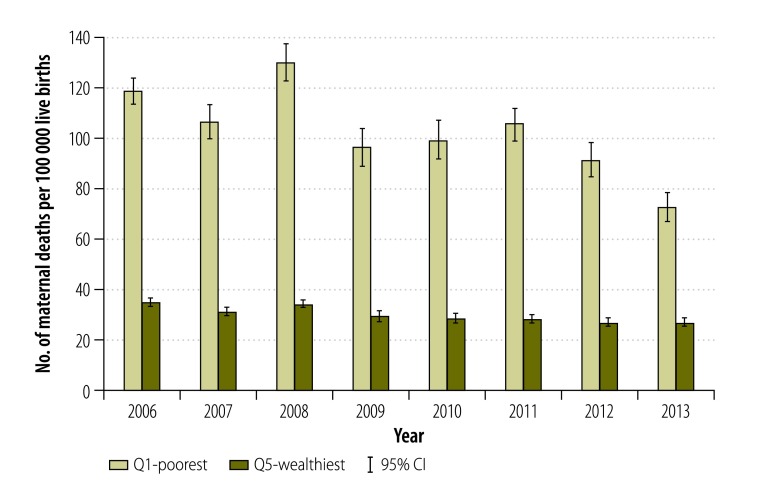
Direct maternal deaths for the wealthiest and poorest quintile, Mexico, 2006–2013

CI: confidence interval; Q: quintile.

Note: Number of direct deaths per 100 000 live births, by year, for the wealthiest and poorest quintile of the municipality-level development index.

**Fig. 4:**
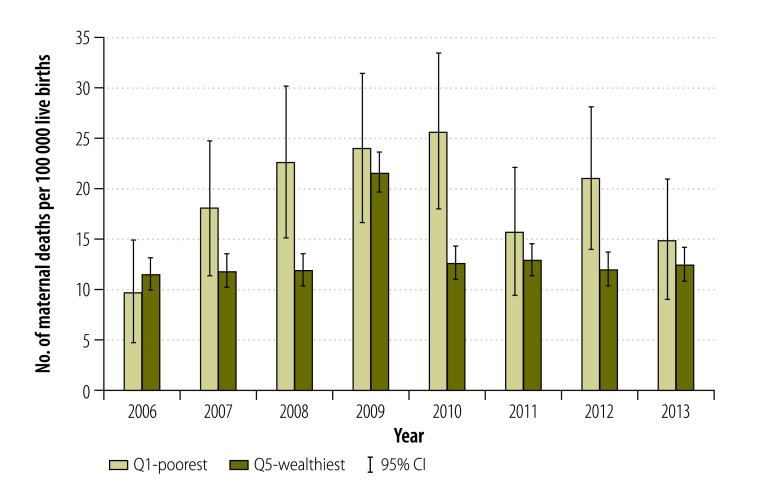
Indirect maternal deaths for the wealthiest and poorest quintile, Mexico, 2006–2013

CI: confidence interval; Q: quintile.

Note: Number of indirect deaths per 100 000 live births, by year, for the wealthiest and poorest quintile of the municipality-level development index.

For direct causes, poorer municipalities had a higher MMR, but also a slightly higher ratio of maternal indirect deaths (Fig. 2). Fig. 3 and Fig. 4 show the MMR by direct and indirect causes between the wealthiest and poorest quintiles by year. Between 2006 and 2013, direct causes of maternal deaths among women residing in the poorest municipalities have nearly halved, going down from 119.1 to 72.7 deaths per 100 000 live births (Fig. 3). The decline in MMR due to direct causes of maternal death in the wealthiest municipalities was 23.5%, going down from 35.2 to 26.9 deaths per 100 000 live births (Fig. 3). For each year and in both poorest and wealthiest quintiles, MMRs for indirect death causes were lower than the MMRs for direct causes. It is only in 2008 and 2010 that the poorest quintile had a statistically significant higher MMR due to indirect causes than the wealthiest quintile (Fig. 4). In 2009, there was a peak in indirect deaths among the wealthiest municipalities, presumably due to the H1N1 epidemic (Fig. 4).

CI: confidence interval.

Note: Pooled number of indirect and direct deaths per 100 000 live births, by quintile of municipality-level development index, 2006–2013.

## Discussion

This paper presents the results of a review and reclassification of causes of maternal death in Mexico, for the period 2006 to 2013. The identified 13% increase in the number of maternal deaths after the BIRMM review suggests that this type of exercise is one approach to correct misclassification of maternal cause-of-death data. There is progress in Mexico towards achieving a reliable assessment of the causes of death in women of reproductive age, with the aim of generating improved data on the causes of maternal mortality.

Our findings show that MMR from direct maternal deaths has been declining between 2006 and 2013; it nearly halved among the poorest women. However, there was no such change in indirect deaths. Given the increase in the burden of noncommunicable diseases and associated risk factors,^30^ indirect deaths may continue to account for an increasing proportion of maternal deaths. Despite the overall decline in MMR from direct deaths, socioeconomic disparities among the poor persist. However, for indirect deaths, women residing in both poor and wealthy areas are affected. These findings are consistent with other studies that examined the obstetric death transition from direct to indirect causes.^36^

Similar projects to the BIRMM have been implemented in other settings as well; however there is a wide variation in the level of maternal death underreporting between settings. Correction factors for the number of maternal deaths range from 1.9 in north-east Brazil^37^^,^^38^ to 3.2 in Menoufia, Egypt.^39^ In high-income countries the correction factor ranges between 0.9 and 2.2.^40^ This observed variation limits our ability to generalize the extent of underreporting or misclassification across settings.

Our review also highlighted the importance of the maternal death review to explain an unusual disease pattern, such as the 2009 H1N1 epidemic in Mexico. This type of temporary change in the pattern of maternal deaths has also been observed in Rwanda and South Africa.^41^ Such increases should be anticipated during an epidemic.

The feasibility of a project that focuses on reclassifying maternal death causes in a given country is dependent on the level of maternal death misclassification or under-coding. In countries with relatively few maternal deaths, extensive review of suspected cases may be feasible. For example, the reproductive age mortality study (RAMOS) investigates all reported deaths in women of reproductive age.^9^^,^^42^ In our study, we applied a cost-saving approach which uses expert opinion to identify a subset of maternal death cases.^26^ Our approach may be more appropriate for countries with poor resources.

Most maternal health interventions are timed around the delivery period of the pregnancy and they focus mainly on skilled birth attendance or emergency obstetric care.^43^ These interventions have an impact on direct deaths and subsequently on the reduction of overall maternal mortality. However, except in cases where complications arise during labour, indirect deaths may not be averted through these delivery-focused interventions.^41^ To reduce indirect deaths, obstetricians and other health-care personnel interacting with pregnant women during the postpartum period need to be trained to treat the entire woman and not just her pregnancy.^44^^,^^45^ Planning for such training requires the development, dissemination and adoption of clinical guidelines.^46^ Effective implementation of such guidelines requires collaboration and the establishment of referral systems between specialities that deal with the major causes of indirect deaths.^41^ For example obstetricians need to be able to effectively communicate with chronic disease specialists regarding at-risk cases.^41^ There is a need for additional surveillance of pregnant women to identify at-risk pregnancies to respond to them appropriately. A health education programme focused on addressing indirect obstetric death risk factors, particularly for women with pre-existing conditions, is needed as well.^46^ Access to and use of effective contraception and safe abortion remains a key strategy to reduce maternal mortality worldwide.^47^

The Mexican Ministry of Health, recognizing the need for quality and state-level maternal mortality estimates, used its authority to ensure cooperation from states for the BIRMM project. While integration of the BIRMM as part of the vital statistics system treats the maternal mortality review as a core public health function,^48^ there is no separate specific budget for it from the ministry of health. The project is being implemented as a non-routine activity, subsidized by committed individuals and interested groups of public health practitioners.^49^ Annual results from the BIRMM are used to adjust official estimates of MMR. The adoption of electronic death registration would allow real-time reporting and validation of suspected maternal deaths.

This study has several limitations. First, the review process did not target all deaths in women of reproductive age, but a subset identified based on the registered cause of death. While the codes used to identify cases capture most misclassified or miscoded maternal deaths, there may be some missed and not investigated.^23^^,^^49^ Second, the review process relies on the availability and quality of additional mortality data sources beyond the death certificate. For some deaths, the additional available information to make a reclassification decision was quite limited. Third, the role of improved ascertainment and categorization must not be overlooked when examining the trends in this analysis. Since the same search procedures have been used each year, it seems unlikely that the observed increase in indirect deaths would be solely due to improved ascertainment. Fourth, a few causes of death, considered to be indirect obstetric causes such as ICD-10 code O26.6 (liver disorders), are subsumed within chapters of the ICD broadly considered for direct maternal deaths. Full ICD-10 codes to the 4-digit level were not available for all deaths so the broader 3-digit categories were used and these cases were considered to be of questionable quality. This means that some rare causes of indirect deaths may have been grouped with direct causes, and if so, the analysis may have underestimated the contribution of indirect deaths. Fifth, missing data in the covariates is another limitation. Sixth, when examining socioeconomic disparities, the use of the municipality-level development index may hide within group differences. It is likely that the individual women dying of maternal deaths across all quintiles of the development index are the poorest women within those municipalities. The area-level nature of this component of the analysis does not allow for commentary about how an individual’s access to resources affects her risk of maternal death.

## Conclusion

This study presents a useful strategy towards achieving a relatively complete and accurate assessment of the causes of maternal mortality in a country with complete vital registration. It provides useful lessons for other countries looking to improve maternal mortality measurement and highlights the importance of developing an appropriate health system response to address indirect maternal deaths.
